# An Unusual Case of Cauda Equina Secondary to Spinal Metastasis of Thyroid Cancer

**Published:** 2016-01

**Authors:** Shabbir Akhtar, Mohammad Adeel

**Affiliations:** 1 *Department of Otorhinolaryngology, Head and Neck Surgery, Aga Khan University Hospital, Karachi, Pakistan. *

**Keywords:** Differentiated thyroid cancer, Follicular thyroid cancer, Metastasis, Spine, Vertebrae

## Abstract

**Introduction:**

Cauda equina secondary to metastatic follicular thyroid cancer of the lumbosacral area is a rare entity.

**Case Report:**

We report an unusual case of a 52-year-old male who presented with backache, lower limb weakness, and perianal numbness. A CT-scan of the lumbosacral area showed an enhancing mass at the L4, L5 and S1 vertebrae. Histopathology after excision revealed a metastatic thyroid cancer. Hence, a CT scan of the neck and chest was performed which showed a nodule in the left lobe of the thyroid and a mass in the left chest wall. A total thyroidectomy and excision of the chest wall lesion was undergone, which was diagnosed as a follicular carcinoma of the thyroid.

**Conclusion:**

Metastatic workup of spinal metastasis should include evaluation of the thyroid gland.

## Introduction

Thyroid cancer is an entity that accounts for approximately 1% of all newly occurring malignant cancers. It accounts for 0.5% of cancers in men and 1.5% in women (1,2). Thyroid cancers are broadly divided into two categories. Differentiated thyroid cancer (DTC) accounts for most malignancies i.e 90% of all thyroid cancers. 

This is further categorized into papillary thyroid cancer (PTC) (70-75%) and follicular thyroid cancer (FTC) (15-20%). Undifferentiated carcinomas, which are anaplastic cancers, account for <5% of thyroid cancers. Medullary carcinoma of the thyroid accounts for 5-10% of thyroid cancers (3,4).

FTC is a slowly growing tumor, which is common in older age groups, with a peak incidence in the fifth decade. Distant metastasis has been reported to occur more commonly in the bones, brain, and lungs (5). The incidence of distant metastasis of FTC has been well documented in literature and is reported to be between11 and 25% (6,7). However, initial presentation of this cancer as distant metastasis, especially in the spine, has yet to be reported.

We present a case of metastatic FTC, whose initial presentation was cauda equina secondary to compression by a metastatic mass in the lumbar region.

## Case Report

A 52-year-old male was presented to the neurosurgery clinic of our hospital with complaints of backache for 3 months, numbness of the legs for 2 months, and weakness of the lower limbs for the last 2 days. Neurological examination revealed a power of 4/5 in both limbs with diminished reflexes, decreased anal tone, and diminished perianal sensation.

**Fig 1:**
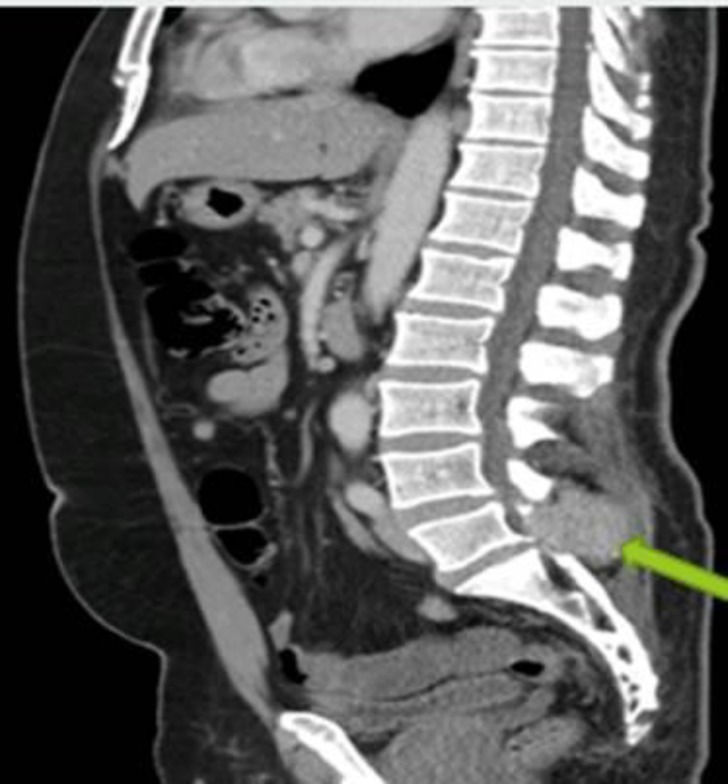
A soft tissue density enhancing expansile lytic lesion involving the spinous process of the L5 vertebra

All laboratory workups, including prostate specific antigen, were within normal limits. Due to the neurological status, an urgent CT-scan of the lumbosacral spine with contrast was carried out, which showed a soft tissue density enhancing expansile lytic lesion (55x44 mm) involving the spinous process of the L5 vertebra that was causing almost complete erosion of the spinous process and part of the lamina of the L5 vertebra (Fig.1).

Further workup was carried out, which included an ultrasound of the abdomen that was reported as normal and a chest x-ray that showed a well defined soft tissue density in the pleura of the left upper lung zone with rib erosion. The patient was sent to the operating room on same day as admission and underwent an L4-L5-S1 Laminectomy, excision of the tumor, and pedicle screw fixation.

**Fig 2:**
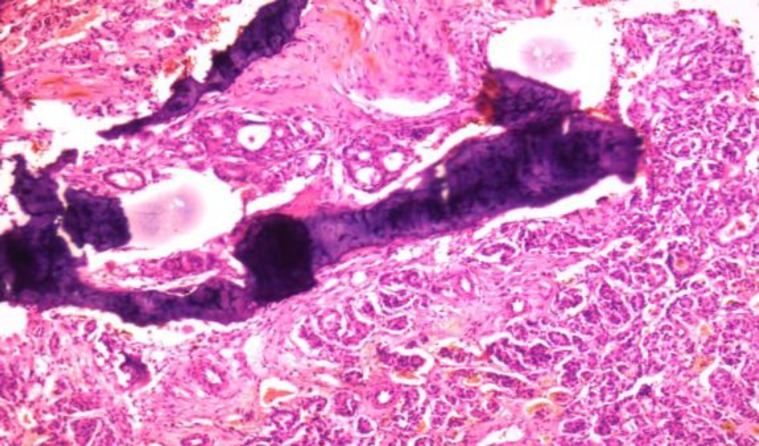
Low power magnification×10; Follicular carcinoma showing multiple variable colloid filled thyroid follicles infiltrating into the darkly stained bony trabeculae (Lumbar specimen

**Fig 3:**
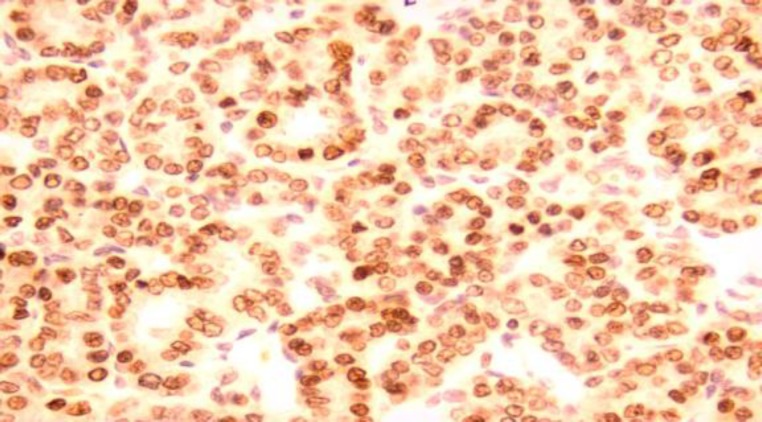
Medium power magnification ×20; Follicular carcinoma cells showing strong nuclear positivity of immunohistochemical marker thyroid transcription factor-1 (TTF-1) confirming thyroidal origin

 Intraoperatively, a mass was observed that was soft to firm in consistency, vascular, and was eroding the spinous process of L4 and L5. Final histopathology revealed a metastatic carcinoma most likely of thyroidal origin as the specimen showed thyroid follicles and was positive for the immunohistochemical marker, Thyroid transcription factor 1 (TTF1) (Figs.2,3). 

**Fig 4:**
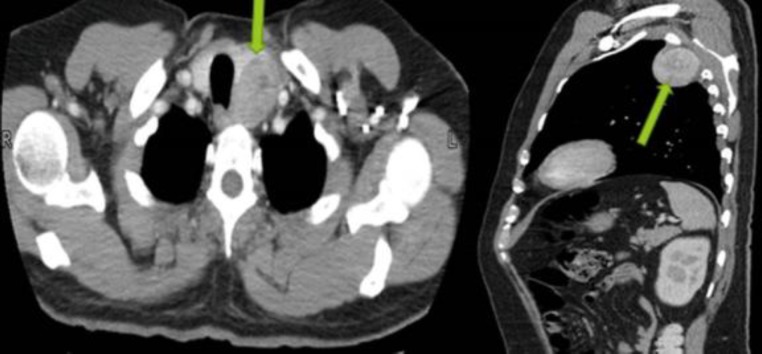
A 4.3 X 3 cm heterogeneously enhancing nodule is identified in the left lobe of the thyroid. An expansile lytic lesion involving the left third rib posterolaterally. is associated with an enhancing partly necrotic soft tissue component abutting the pleura and measuring 5 X 4 cm

Therefore, serum thyroglobulin marker and thyroid profile were carried out. Only thyroglobulin levels were raised i.e 103 (normal <55ng/ml). A CT-scan of the neck and chest was also carried out, which showed a heterogeneously enhancing nodule in the left lobe of the thyroid. It measured 4.3 X 3 cm and another expansile lytic lesion involving the left third rib posterolaterally was also seen measuring 5 X 4 cm (Fig.4).

**Fig5:**
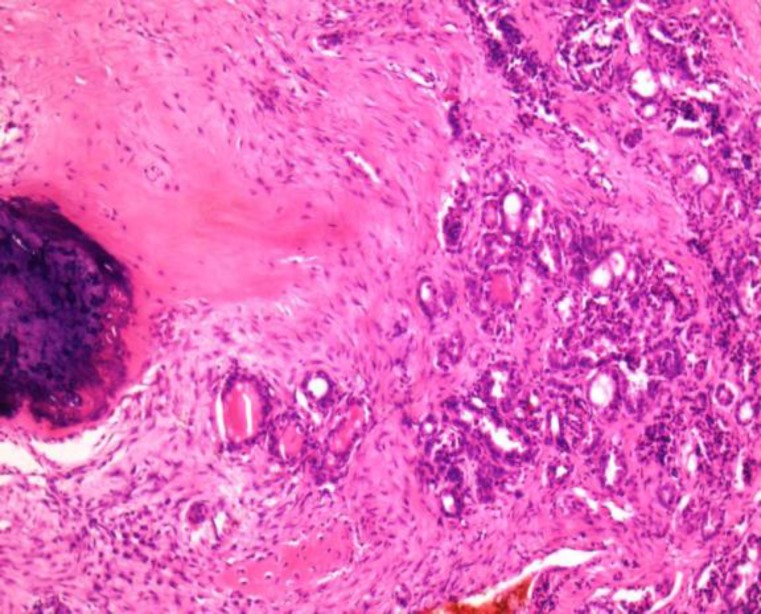
Low power magnification×10; Follicular carcinoma showing multiple variable colloid filled thyroid follicles infiltrating into the darkly stained bony trabeculae (rib specimen

The patient then underwent total thyroidectomy and left thoracotomy with excision of the mass along with the third rib. Final histopathology revealed a follicular carcinoma in the left thyroid and metastatic follicular carcinoma in the 3^rd^ rib mass (Fig.5).

The patient was then given 200mci of radioiodine and a post ablative scan was performed that showed minimal uptake in the lumbar region so another dose of 200mci was given. Repeat thyroglobulin showed a decline from 103 to 13.8ng/ml.

The patient slowly regained power in his limbs with aid of physiotherapy and bowel habits returned to normal. At his one year of follow up he showed to be symptom free and to have resumed his job.

## Discussion

The most common initial presentation of thyroid malignancies is a thyroid nodule (90%) followed by cervical lymphadeno- pathy (5%) and the rest in the lung, bone, liver, etc…(2,8). The incidence of bone metastasis in DTC is 2-13%. FTC has a higher incidence of bone metastasis of 7–20% as compared to PTC, which has an incidence of spinal metastasis of 1–7%(9,10). The 10-year survival rate in DTC is 80-95%. However, this figure drops to about 40% when distant metastasis is present (11).

FTC is known for its hematogenous spread secondary to vascular invasion. The most frequent site that it can metastasize to is the lung followed by bone and lymph nodes (7,12). Bone metastasis is more frequent in FTC and has been reported in 7–20% of cases compared to PTC where bone metastasis occurs in 1.4-7% of cases (2). McCormack however, has reported thyroid cancer metastasis to bone varying from as low as less than 1% to more than 40% (13). 

In literature, there are few reports that have described distant metastasis as the initial presentation of thyroid cancer. Shaha et al (6), from Sloan Kettering Memorial Hospital, reported the incidence of distant metastasis to be 11% in 1038 patients with thyroid cancer, in which 4% of patients had an initial presentation of distant metastasis.

Complete surgical removal of the tumor has been reported to offer prolonged survival (12). Demura et al (14), in their study on 10 patients suffering from thyroid cancer with spinal metastasis, showed a survival rate of 74% and 25% after 5 and 10 years respectively via en-bloc resection. Local recurrence was reported to be high i.e 57% in cases who underwent debulking compared to a recurrence rate of 10% in cases who underwent total en bloc spondylectomy. Similarly a retrospective study conducted by Bernier et al (15), on 109 patients among which 68% had spinal metastasis, showed that complete spinal metastasis surgery on multivariate analyses was an independent prognostic indicator of improved survival. In our patient, the metastatic tumor was completely removed from the spine followed by total thyroidectomy and complete excision of the chest wall metastasis followed by radioiodine. After his one-year follow up, our patient is well, symptom free, and has resumed his job. 

## Conclusion

Diagnosis of metastatic tumors of the vertebrae need a thorough work up that should include assessment of the thyroid gland entailing a detailed clinical history and a physical examination. In conclusion, for these kinds of rare cases, early presentation, proper examination, early diagnosis, prompt initiation of treatment and follow-up can possibly prolong the patient’s life and improve quality of life.

## References

[1] Sherma SI (2003). Thyroid carcinoma. The Lancet.

[2] Muresan M, Olivier P, Leclère J, Sirveaux F, Brunaud L, Klein M, Zarnegar R, Weryha G (2008). Bone metastases from differentiated thyroid carcinoma. Endocrine-related cancer.

[3] Wilson P, Millar B, Brierley J (2004). The management of advanced thyroid cancer. Clinical Oncology.

[4] Sciubba DM, Petteys RJ, Kang S, Than KD, Gokaslan ZL, Gallia GL, Wolinsky J-P (2010). Solitary spinal metastasis of Hürthle cell thyroid carcinoma. Journal of Clinical Neuroscience.

[5] Rodrigues G, Ghosh A (2003). Synchronous bony and soft tissue metastases from follicular carcinoma of the thyroid. Journal of Korean Medical Science.

[6] R Shaha A, P Shah J, R Loree T (1997). Differentiated thyroid cancer presenting initially with distant metastasis. The American journal of surgery.

[7] Girelli M, Casara D, Rubello D, Piccolo M, Piotto A, Pelizzo M, Busnardo B (1993). Metastatic thyroid carcinoma of the adrenal gland. Journal of endocrinological investigation.

[8] Hindié E, Zanotti-Fregonara P, Keller I, Duron F, Devaux J-Y, Calzada-Nocaudie M (2007). Bone metastases of differentiated thyroid cancer: impact of early 131I-based detection on outcome. Endocrine-related cancer.

[9] Wexler JA (2011). Approach to the thyroid cancer patient with bone metastases. Journal of Clinical Endocrinology and Metabolism.

[10] Durante C, Haddy N, Baudin E, Leboulleux S, Hartl D, Travagli J (2006). Long-term outcome of 444 patients with distant metastases from papillary and follicular thyroid carcinoma: benefits and limits of radioiodine therapy. Journal of Clinical Endocrinology & Metabolism.

[11] Eustatia-Rutten C, Romijn J, Guijt M, Vielvoye G, Van den Berg R, Corssmit E (2003). Outcome of palliative embolization of bone metastases in differentiated thyroid carcinoma. Journal of Clinical Endocrinology & Metabolism.

[12] Kiml Y-S (2002). Metastatic follicular thyroid carcinoma to the thymus in a 35-year-old woman. Yonsei medical journal.

[13] McCormack KR (1966). Bone metastases from thyroid carcinoma. Cancer.

[14] Demura S, Kawahara N, Murakami H, Abdel-Wanis ME, Kato S, Yoshioka K (2011). Total en bloc spondylectomy for spinal metastases in thyroid carcinoma: Clinical article. Journal of Neurosurgery: Spine.

[15] Bernier M-O, Leenhardt L, Hoang C, Aurengo A, Mary J-Y, Menegaux F (2001). Survival and therapeutic modalities in patients with bone metastases of differentiated thyroid carcinomas. Journal of Clinical Endocrinology & Metabolism.

